# Promoting Conversations About Cognitive Decline Between Older Adults and Primary Care Providers

**DOI:** 10.1093/geroni/igaa057.513

**Published:** 2020-12-16

**Authors:** Benjamin Olivari, Christopher Taylor, Nia Reed, Lisa McGuire

**Affiliations:** 1 CDC, Atlanta, Georgia, United States; 2 Centers for Disease Control and Prevention, Atlanta, Georgia, United States

## Abstract

Alzheimer’s disease and related dementias often begin with symptoms of mild memory loss, eventually leading to more severe cognitive impairment, functional impairment, and ultimately, death. Data from the Behavioral Risk Factor Surveillance System core questions related to chronic diseases and from the cognitive decline optional module on subjective cognitive decline (SCD) from the years 2015-2018 were aggregated across the participating 50 states, D.C., and Puerto Rico for this analysis. Among U.S. adults aged 65 years and older, only 39.8% (95%CI=37.6-42.1) of those experiencing SCD reported discussing their SCD symptoms with a healthcare provider. The prevalence of discussing SCD symptoms with a provider was higher among those with at least one chronic condition than among those with no chronic conditions. 30.7% (28.6-32.8) of those aged 65 years and older reported that their SCD led to functional limitations and 28.8% (26.5-31.2) needed assistance with day-to-day activities. For patients aged 65 years and older, Welcome to Medicare visits and Medicare Annual Wellness Visits are critically underutilized primary care access points. Primary care providers can manage chronic conditions, cognitive health, and initiate referrals for testing. Efforts to promote the use of toolkits and diagnostic codes that are available to primary care providers to initiate conversations about memory loss with patients may be utilized to improve detection, diagnosis, and planning for memory problems. Discussions may lead to earlier detection and diagnosis of cognitive impairment, such as Alzheimer’s disease, or other treatable conditions such as delirium or pressure in the brain and avoid costly hospitalizations.

